# Supplementary material for: Regulation of the activity of the bacterial histidine kinase PleC by the scaffolding protein PodJ
Source: J Biol Chem. 2022 Feb 3;298(4):101683. doi: 10.1016/j.jbc.2022.101683 (PMC8980812; doi:10.1016/j.jbc.2022.101683)
Supplement: Supplemental Figures S1–S10, Tables S1–S4 and Text S1 [file mmc1.pdf]

Supplementary Information for

Regulation of the activity of the bacterial histidine kinase PleC by scaffolding protein PodJ

Chao Zhang[1], Wei Zhao[1], Samuel W. Duvall[1], Kimberly A. Kowallis[1], W. Seth Childers[1]

[1]Department of Chemistry, University of Pittsburgh, Pittsburgh, PA 15260, USA.

This PDF file includes:

- Figures S1 to S10
- Tables S1 to S4
- Text S1
- SI References

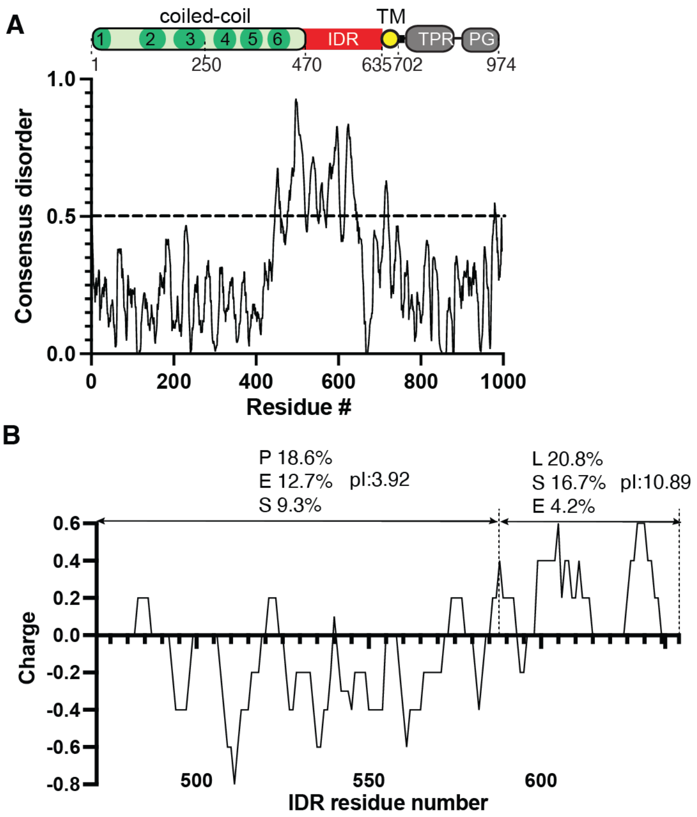

Figure S1. **PodJ domain architecture, IDR charge and composition analysis.** (A)PodJ domain organization predicted by HHpred and adapted from previous studies[1,2] The coiled-coil rich region was analyzed by PCOILS[3] and modeled with MODELLER[4]. The probability of intrinsic disorder over the primary sequence of PodJ represents disorder prediction from algorithm Metapredict[5]. Dash line at 0.5 on the y axis indicates the threshold for disorder probability. (B) Charge analysis of PodJ IDR in a window of 5 residues. The top 3 charged residues, their percentages and calculated pI for each block are listed.

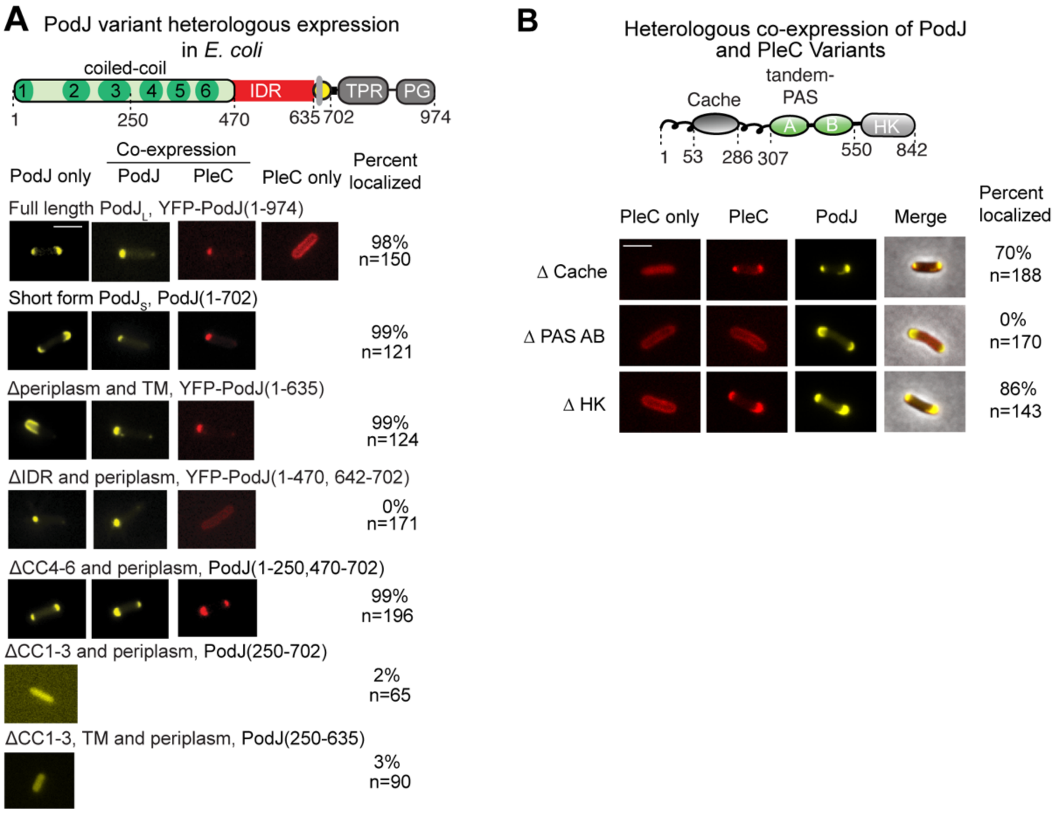

Figure S2: Heterologous expression of PodJ and PleC variants to identify sites of interaction. (A) Heterologous expression of a set of YFP-PodJ domain deletion variants alone or co-expressed with PleC-mCherry in *E. coli*. YFP-PodJ variants were induced with 0.5 mM IPTG, and PleC-mCherry was induced with 1mM arabinose for 2 hours. Scale bar: 2 μm. (B) Heterologous co-expression of PleC-mCherry variants with YFP-PodJ in *E. coli*. Scale bar: 2 μm. YFP-PodJ was induced with 0.5 mM IPTG, and PleC-mCherry was induced with 1 mM arabinose for 3 hours.

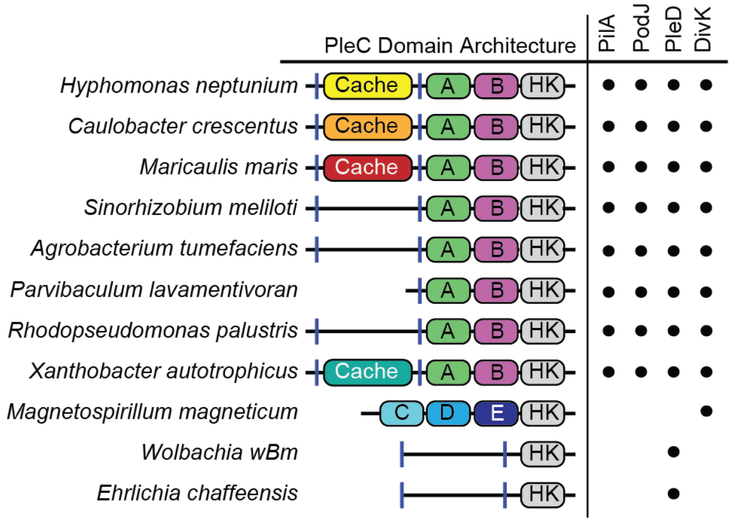

Figure S3. **PodJ's interaction with PleC through PAS A and B domains is conserved in other alpha-proteobacteria.** PleC domain architecture from selected alpha-proteobacteria and conservation of PleC specific inputs and cognate response regulators. Domains A and B represent both cytoplasmic PAS domains of PleC.

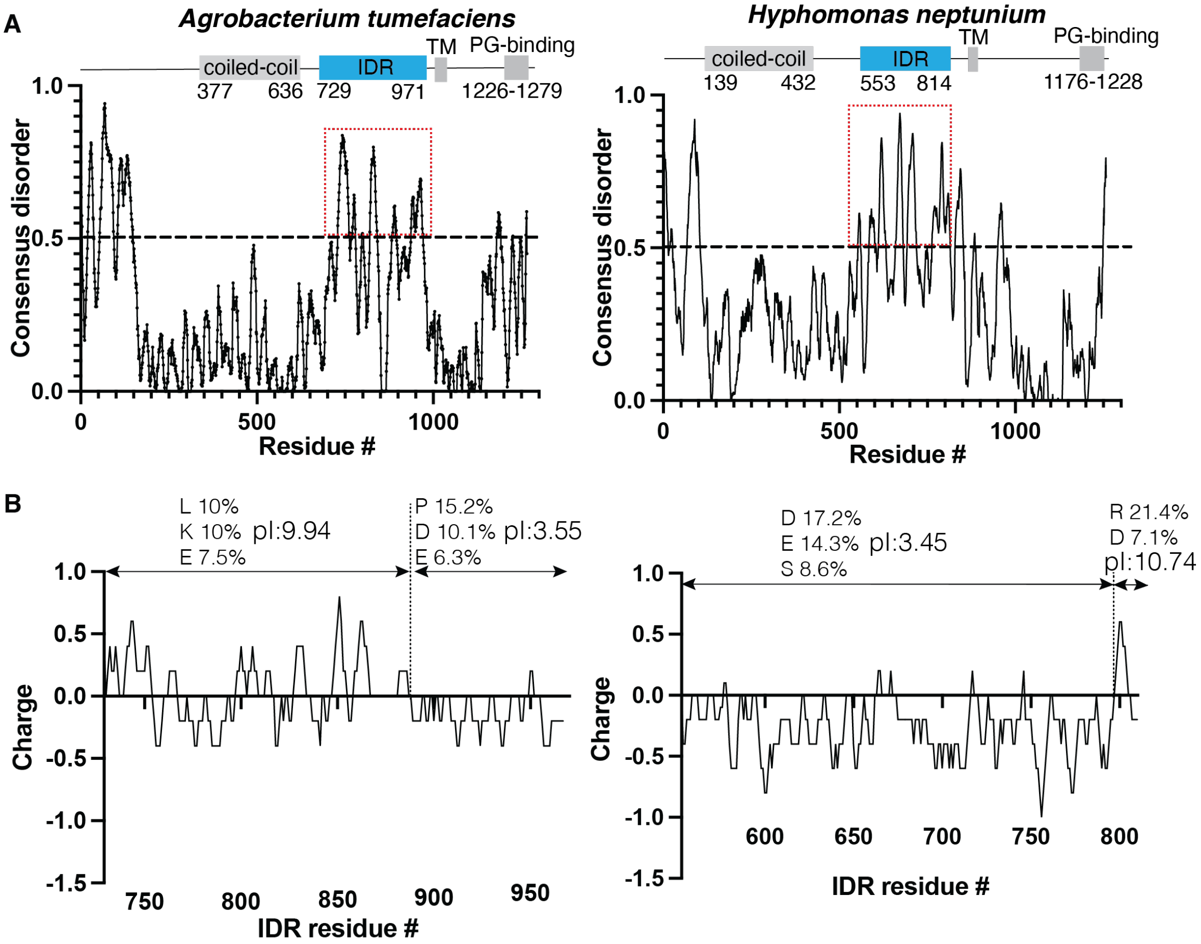

Figure S4. **PodJ homologs domain architecture, IDR, charge, and composition analysis from two selected alphaproteobacteria.** (A) Domain architecture and intrinsically disordered region prediction of PodJ homologs. The coiled-coil region was predicted from UniProt[6]. Transmembrane domains were predicted using TMPred[7]. Intrinsically disordered regions were predicted using metapredict[5]. Residues with disorder probability greater than 0.5 were shown in the rectangular dash red box. (B) Charge and compositional analysis of IDR in PodJ homologs. Amino acid composition and pI were calculated using EMBOSS[8]. The dominant charged residues and calculated pI for each block were also shown.

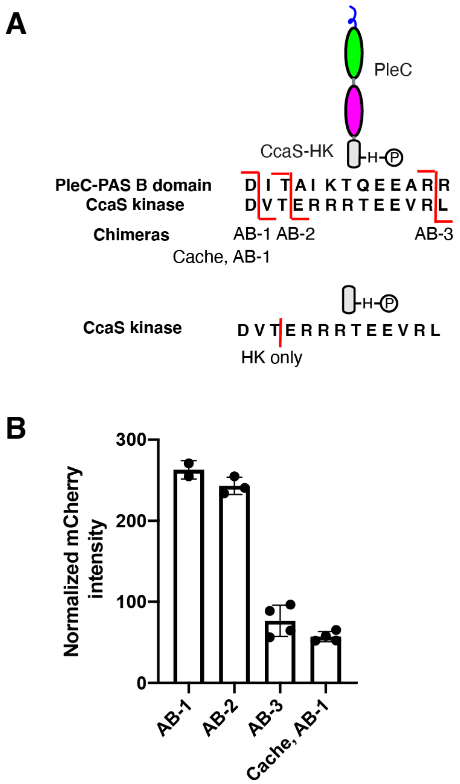

Figure S5. **Design and screening of PleC-CcaS chimeras that respond to PodJ expression.** (**A**). Design of PleC-CcaS chimera from light-sensing kinase CcaS and PleC. Sequence alignment between PleC PAS B and CcaS HK suggests a homology region exists after the DI/VT hinge motif. An HK-only construct without sensory domains was used as a negative control. AB indicates PleC PAS AB was used for fusion protein, while Cache AB suggests the full sensory region of PleC was used for chimera construction. (**B**). Screening of four PleC-CcaS chimeras for responsiveness to PodJ expression.

A

```
PleC_PAS_A      KAEVAHREFIDSERRFRLAVEAARCGIWEWDLNGDQVYLSDVTGAMFGWGGGGVVSGQDL   60
PleC_PAS_B      EERIAQARAQAAENRLRDAIESVSEAFVLWDRQGRLLMCNRNYRSVFSLEPKILKPGAA-   59
                : .:*: .    :*.*:* *:*:.   .:  ** :*  :   .     ::*.      :  *

PleC_PAS_A      LERISIDHRERVRQALANAAMYGAFDVSFRVPA--SEQGA------RSLWIDARGQGFGK   112
PleC_PAS_B      ---------------RAEVNRFAALAIKQDHPAPDGAKGVREAEMMDGRWIQISERRTAE   104
                               *:.  :.*: :.   **  . :*.        . **:   :  .:

PleC_PAS_A      PGSEGHARIIGVALDVT         129
PleC_PAS_B      GG------LVMTAADIT         115
                 *      :: .* *:*
```

B

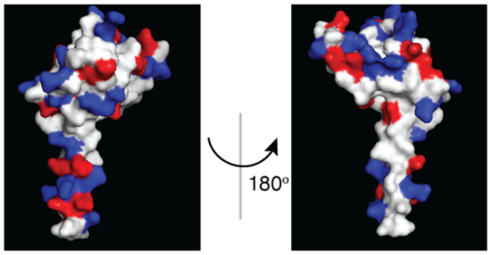

C

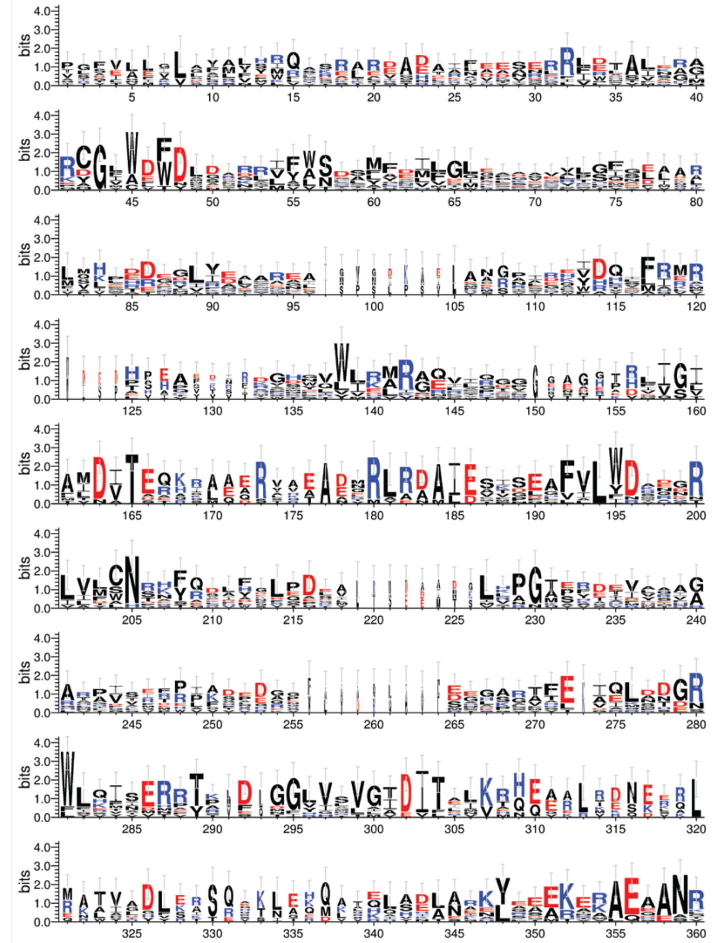

D

PAS A BLAST against alphaproteobacteria

Accession	Per. Ident	Evalue
HEV42420.1	76.74	5.13E-56
MBA4013983.1	73.64	1.29E-52
MBP6878882.1	74.42	4.00E-51
WP_156378276.1	74.42	4.11E-51
KQW72874.1	74.42	4.34E-51
WP_183774560.1	74.42	5.77E-51
WP_215341570.1	69.77	8.27E-50
ODU33946.1	73.64	8.96E-50
THD65268.1	68.22	2.59E-49
WP_056733077.1	73.64	2.74E-49
MBT9472795.1	73.64	2.74E-49
MBP7649181.1	68.99	5.37E-49
MBP6545846.1	68.99	7.39E-49
THD59163.1	66.67	8.72E-49
RYF94744.1	75.21	2.00E-48
THD79946.1	65.89	1.38E-47
WP_012523228.1	65.12	2.03E-47
MBU2134991.1	72.87	5.25E-47
WP_111513056.1	65.89	8.72E-47
MBI5942540.1	71.32	2.17E-46
MAK81792.1	72.87	4.00E-46
WP_111456050.1	64.34	1.07E-45
WP_111530019.1	66.14	2.69E-45
OYX31829.1	61.60	3.57E-43
OYU70959.1	66.67	4.38E-43
MBI1196418.1	64.71	6.66E-43
MBA4792159.1	68.99	1.07E-42
MBU1376023.1	62.50	3.54E-42
MBL8773636.1	63.03	4.24E-42
PZQ63279.1	61.67	4.99E-42
WP_111276259.1	63.03	6.35E-42
OXE37280.1	67.44	1.26E-41
WP_156466929.1	61.67	2.33E-41
TAJ70067.1	62.19	2.92E-41
MBJ7411368.1	61.67	2.96E-41
OHB27668.1	61.67	3.28E-41
Average	**68.28**	

PAS B BLAST against alphaproteobacteria

Accession	Per. Ident	E value		Accession	Per. Ident	E value
HEV42420.1	91.30	3.47E-58		PZU56446.1	57.39	1.65E-35
OYW90456.1	85.22	3.88E-54		WP_207870595.1	54.78	1.66E-35
MBT9472795.1	85.22	1.01E-53		EGF96315.1	54.78	2.61E-35
WP_056733077.1	85.22	1.19E-53		EDK81457.1	56.52	2.63E-35
RYF94744.1	85.22	1.24E-53		PZO08317.1	56.52	2.82E-35
MAK81792.1	86.09	1.23E-52		WP_081836168.1	56.52	2.82E-35
MBI5942540.1	81.74	1.88E-51		WP_183196426.1	56.52	2.89E-35
MBA4013983.1	82.61	1.99E-51		MBB3872164.1	56.52	2.93E-35
WP_183774560.1	82.61	1.99E-51		OGN47325.1	56.52	3.26E-35
WP_156378276.1	82.61	2.25E-51		MBJ7317341.1	56.52	3.34E-35
KQW72874.1	82.61	2.30E-51		KDP94631.1	56.52	3.36E-35
MBP7649181.1	83.48	2.41E-51		WP_040349769.1	56.52	3.36E-35
MBP6545846.1	83.48	3.52E-51		OGN48782.1	56.52	3.36E-35
ODU33946.1	82.61	4.09E-51		MBU1273609.1	56.52	3.41E-35
TAJ70067.1	73.04	5.18E-51		WP_196066342.1	56.52	3.46E-35
WP_111530019.1	73.04	6.81E-51		RYG16086.1	53.04	3.51E-35
WP_215341570.1	82.61	9.66E-51		HAF79465.1	54.37	3.69E-35
MBL8554656.1	70.44	5.61E-50		TAJ56292.1	54.78	4.50E-35
PZQ63279.1	72.17	7.69E-50		WP_090647314.1	56.52	5.21E-35
MBP6878882.1	80.87	8.37E-50		SCW53406.1	56.52	6.21E-35
WP_111276259.1	71.30	1.96E-49		MBI2261230.1	55.65	7.93E-35
MBJ7411368.1	72.17	2.13E-49		MBA3049353.1	54.78	1.12E-34
MBU1376023.1	71.30	2.18E-49		WP_107871417.1	56.52	1.23E-34
OHB27668.1	72.17	3.29E-49		WP_013479453.1	58.26	1.26E-34
WP_156466929.1	71.30	3.44E-49		WP_126421283.1	58.26	1.27E-34
MBL8773636.1	70.44	1.05E-48		BBF80745.1	58.26	1.27E-34
MBA4792159.1	81.74	1.50E-48		WP_183203771.1	55.65	1.48E-34
WP_012523228.1	71.30	1.15E-47		RZJ29588.1	53.15	1.92E-34
MBQ1542868.1	79.13	1.52E-47		WP_123287820.1	56.52	2.60E-34
WP_111456050.1	73.04	3.01E-47		WP_040344694.1	54.78	3.32E-34
OYX31829.1	71.30	4.09E-47		OGN50218.1	55.65	3.82E-34
THD59163.1	78.26	4.66E-47		MBB4082137.1	55.65	4.09E-34
ODT85277.1	69.57	8.90E-47		WP_087140987.1	54.78	4.30E-34
THD65268.1	75.65	2.14E-45		HAV50066.1	56.52	4.31E-34
MBI1196418.1	67.83	1.07E-44		MAL88326.1	56.52	4.62E-34
OYU70959.1	73.04	8.39E-42		OYX21121.1	54.78	5.23E-34
THD79946.1	63.48	1.33E-40		WP_076223497.1	54.78	5.88E-34
MBE7219139.1	64.91	3.07E-39		WP_128719619.1	54.78	5.88E-34
WP_168045073.1	59.13	1.46E-38		MBD3817583.1	54.78	5.99E-34
WP_111513056.1	68.70	2.91E-38		WP_088491656.1	54.78	6.17E-34
MBF0665016.1	60.00	7.01E-38		WP_088411220.1	54.78	6.23E-34
WP_029087896.1	60.87	2.01E-37		WP_128115884.1	54.78	7.28E-34
MBJ7485572.1	57.39	2.28E-37		ESQ86095.1	56.52	9.96E-34
NBW09018.1	55.65	2.21E-36		WP_081714526.1	56.52	1.12E-33
MBU2271017.1	56.52	2.53E-36		WP_178827671.1	54.78	1.33E-33
MBU1383432.1	56.52	2.73E-36		WP_114817891.1	54.78	1.39E-33
MBU2417434.1	56.52	2.99E-36		MBD3833676.1	54.78	1.50E-33
WP_183254730.1	57.39	5.87E-36		WP_141628378.1	53.04	1.60E-33
MBN8552289.1	59.13	7.85E-36		WP_023460854.1	55.65	1.71E-33
OXE37280.1	68.70	1.14E-35				
MBD3835210.1	57.39	1.16E-35		Average	**64.44**	

Figure S6. **Bioinformatic analysis of PleC.** (**A**). Sequence similarity analysis of alignment PleC PAS A and B domains (**B**). Illustration of surface-exposed charged residues of PleC PAS B domain homology model. Color code: white (neutral), red (negative) and blue (positive). (**C**). Sequence logo[9] of PleC PAS AB domain from residue 290-580. (D) Sequence similarity analysis of PleC PAS A and B domains. PleC PAS A and B domains were searched in BLAST against alphaproteobacteria, and the average sequence similarity was calculated for each domain.

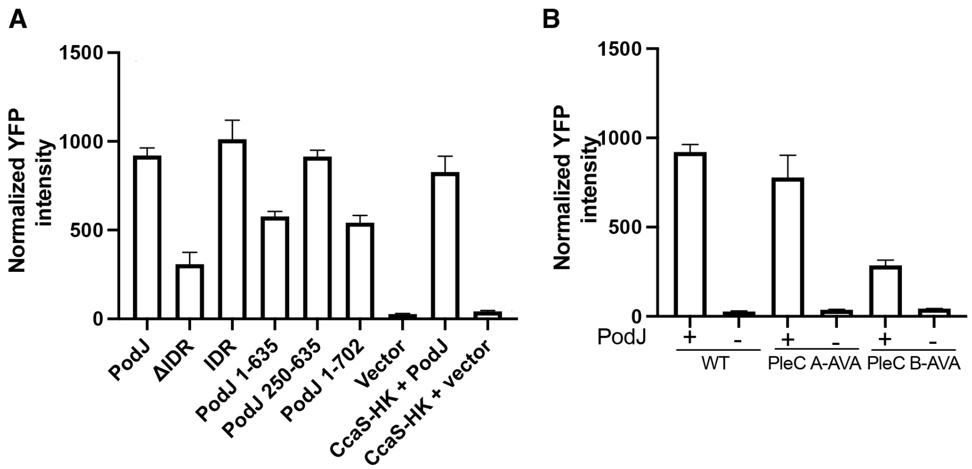

Figure S7. Quantification of YFP-PodJ and its variant expression level in PleC-CcaS reporter system assays in *E. coli.* (A). The expression level of YFP-PodJ variants (B). The expression level of YFP-PodJ in PleC PAS A and B signal transmission DI/VT motif and mutants.

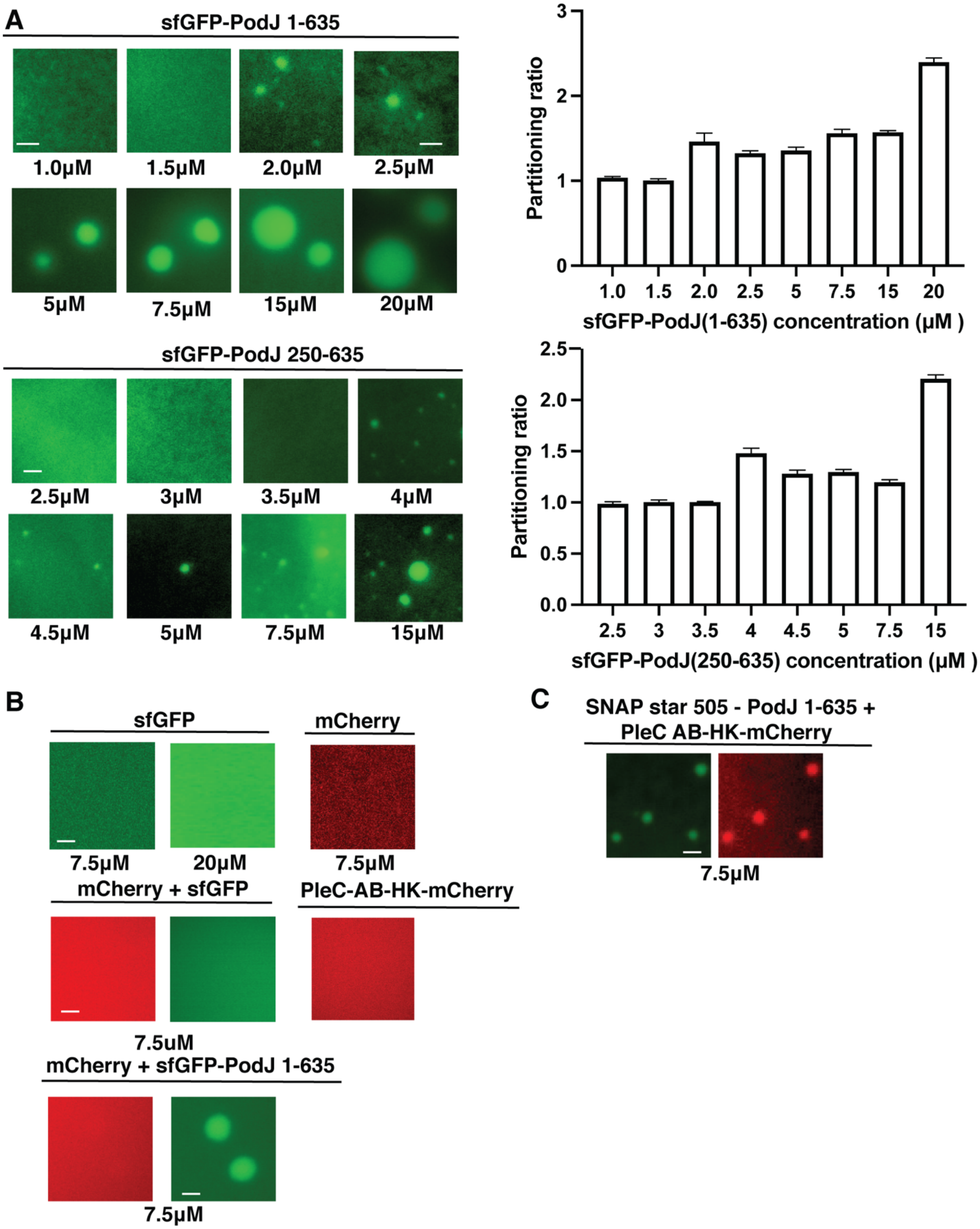

Figure S8. **PodJ's ability to form biomolecular condensates depends on coiled-coil 1-3 region but not fluorescent protein.** (A). Concentration dependency of sfGFP-PodJ1-635 and sfGFP-PodJ250-635 phase separation. Scale bar: 2.0 μm. (B) PodJ

recruits PleC into biomolecular condensates independent of fluorescent proteins. sfGFP, mCherry, mCherry plus sfGFP did not phase separate at low PEG8000 concentrations. PleC AB-HK only forms biomolecular condensates at relatively high PEG8000 concentrations. mCherry was not recruited by sfGFP-PodJ 1-635 at various PEG 8000 concentrations. Scale bar: 2.0 µm. (C) Swapping sfGFP to SNAP-tag retained PodJ's droplet formation as well as PleC AB-HK-mCherry recruitment capability. All samples were prepared in 50 mM Tris-HCl, pH=8.0, 200mM KCl with varying amounts of proteins unless otherwise specified at room temperature. Representative images were shown from the whole field of imaging. Scale bar: 2.0 µm.

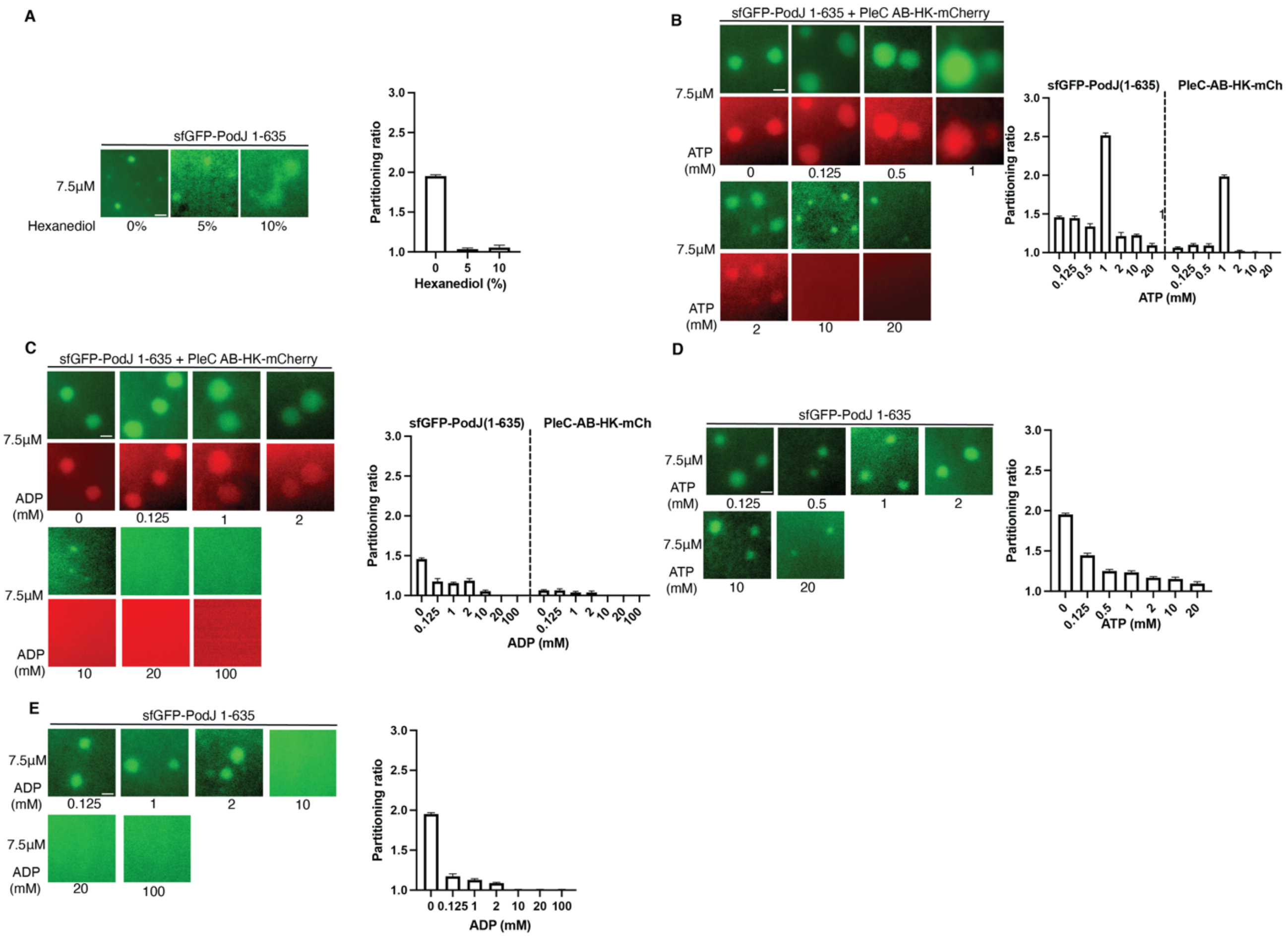

Figure S9. PodJ biomolecular condensates material properties are regulated by hexanediol, ATP or ADP addition *in vitro*. (A). Hexanediol disrupts well-organized droplet formation of sfGFP-PodJ 1-635 in a dose-dependent manner. Scale bar: 2.0 µm. (B). The addition of ATP regulates droplet formation and dissolution of the sfGFP-PodJ 1-635 and PleC AB-HK-mCherry complex in the droplets in a dose-dependent manner. Scale bar: 2.0 µm. (C) The addition of ADP regulates droplet formation and dissolution of the sfGFP-PodJ 1-635 and PleC AB-HK-mCherry complex in the droplets in a dose-dependent manner. Scale bar: 2.0 µm. (D) ATP acts as a hydrotrope that leads to the dissolution of droplets at high concentrations. Scale bar: 2.0 µm. (E) ADP acts as a hydrotrope that leads to the dissolution of droplets at high concentrations. Scale bar: 2.0 µm.

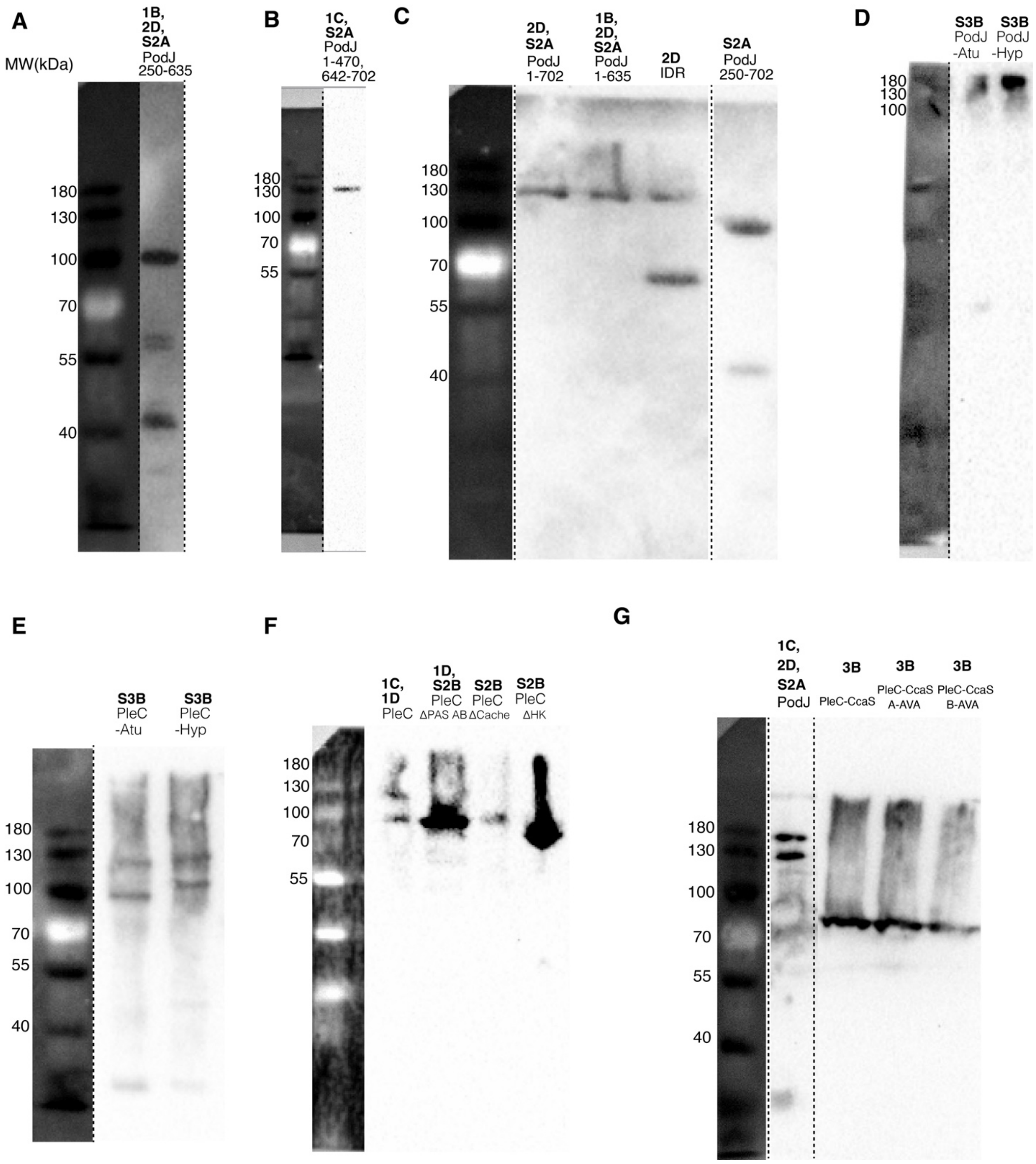

Figure S10 Western blotting for constructs used in this work. (A)(B)(C) YFP/sfGFP-PodJ variants from *C. crescentus* expressed in *E. coli* or *C. crescentus* and from alphaproteobacteria species expressed in *E coli*. Blots spliced from the same gel were marked by a dashed black line. (D) YFP-PodJ from alphaproteobacteria species

expressed in *E coli*. (E). CFP-PleC from alphaproteobacteria species expressed in *E coli*. (F) PleC-mCherry variants from *C. crescentus* expressed in *E. coli* or *C. crescentus.* (G). YFP-PleC-CcaS signal transmission motif wild type and mutants expressed in *E coli.* The figure that each construct corresponds to is listed on top of each construct name in bold. Full-length PodJ constructs, even when expressed in *E. coli*, exhibit proteolysis into a shorter form of PodJ. In addition, PodJ(250-635) may exhibit proteolysis or non-specific binding in the western blot assay.

Table S1. List of PCR reactions for Gibson assembly

Plasmid	First Insert Forward Primer	First Insert Reverse Primer	Second Insert Forward Primer	Second Insert Reverse Primer	Third Insert Forward Primer	Third Insert Reverse Primer
pWZ21	WZ0044_(Plec)_forward	WZ0045_(Plec)_reverse	WZ0046_(mcherry)_forward	WZ0024_(mcherry)_reverse		
pWZ24	WZ0050_(PBAD)_forward	WZ0051_(PBAD)_reverse	WZ0052_(YFP-PODJ)_forward	WZ0053_(YFP-PODJ)_reverse		
pWZ12	WZ0025_(pxyfpc-6)_forward	WZ0010_(PCDF-YFP)_reverse	WZ0011_(HRSAT)_(PODJ)_forward	WZ0026_(PodJ)_reverse		
pWZ138-kan	WZ0324_(pbvmcs-6)_forward	WZ0325_(pbvmcs-6)_reverse	WZ0326_(plec-mch)_forward	WZ0327_(plec-mch)_reverse		
pWZ138-chlor	WZ0324_(pbvmcs-6)_forward	WZ0325_(pbvmcs-6)_reverse	WZ0326_(plec-mch)_forward	WZ0327_(plec-mch)_reverse		
pCZ340	CZ10807_(pCDF)_forward	CZ10808_(pCDF)_reverse	CZ10809_(Atu_PleC)_forward	CZ10810_(Atu_PleC)_reverse		
pCZ341	CZ10811_(pCDF)_forward	CZ10812_(pCDF)_reverse	CZ10813_(Xan_PleC)_forward	CZ10814_(Xan_PleC)_reverse		
pCZ342	CZ10815_(pCDF)_forward	CZ10816_(pCDF)_reverse	CZ10817_(Hyp_PleC)_forward	CZ10818_(Hyp_PleC)_reverse		
pWZ127	WZ0173_(pbad)_forward	WZ0312_(pbad)_reverse	WZ0315_(plec-pasc)_forward	WZ0316_(plec-pasc)_reverse	WZ0317_(mcherry)_forward	WZ0176_(plec-mcherry)_reverse
pWZ135	WZ0173_(pbad)_forward	WZ0322_(pbad)_reverse	WZ0323_(plec-pasd-atg)_forward	WZ0320_(plec-pasd)_reverse	WZ0321_(mcherry)_forward	WZ0176_(plec-mcherry)_reverse
pWZ136	WZ0173_(pbad)_forward	WZ0312_(pbad)_reverse	WZ0315_(plec-pasc)_forward	WZ0320_(plec-pasd)_reverse	WZ0321_(mcherry)_forward	WZ0176_(plec-mcherry)_reverse
YFP-J23102-pCZ267	CZ10625_(pCZ10224)_forward	CZ10622_(Delta_CcaS)_reverse	CZ10623_(eyfp)_forward	CZ10626_(eyfp)_reverse		
pCZ259	CZ10596_(pCZ130)_forward	CZ10597_(pCZ130)_reverse	CZ10598_(CloDF13)_forward	CZ10599_(CloDF13)_reverse		
pCZ253	CZ10583_(CcaS_with_TM)_forward	CZ10524_(pSR43_6)_reverse	CZ10525_(PleC_PAS3)_forward	CZ10584_(PleC_CD)_reverse		
pW173	WZ0050_(PBAD)_forward	WZ0186_(pcdf-mcherry)_reverse	WZ0187_(PODJ1-470)_forward	WZ0446_(podjdeltaPSE)_reverse		
pCZ412	CZ11031_(pBAD-YFP)_forward	CZ11032_(pBAD-YFP)_reverse	CZ11033_(PSE)_forward	CZ11034_(PSE)_reverse		
pCZ253 (D433A, T435A)	CZ-pCZ253-DVT-C-AVA-F	CZ-pCZ253-DVT-C-AVA-R				
pCZ253 (D548A, I549V, T550A)	CZ11057_(pWZ21)_forward	CZ11058_(pWZ21)_reverse	CZ11059_(PleC_CD_D_DVT)_forward	CZ11060_(PleC_CD_D_DVT)_reverse		
pCZ253 (D433A, T435A, D548A, I549V, T550A)	CZ11057_(pWZ21)_forward	CZ11058_(pWZ21)_reverse	CZ11059_(PleC_CD_D_DVT)_forward	CZ11060_(PleC_CD_D_DVT)_reverse		
pCZ391	CZ10973_(pTEV5)_forward	CZ10974_(pTEV5)_reverse	CZ10975_(msfGFP)_forward	CZ10976_(msfGFP)_(stop)_reverse		
pmas00001	mas00001_(pTEV5)_forward	mas00002_(pTEV5)_reverse	mas00003_(mCherry)_forward	mas00004_(mCherry)_reverse		
pCZ388	CZ10944_(pTEV5-bb)_forward	CZ10964_(pTEV5-bb)_(S)_reverse	CZ10965_(msfGFP)_forward	CZ10966_(msfGFP)_(HRSAT)_reverse	CZ10948_(PodJ-1-635)_forward	CZ10949_(PodJ-1-635)_reverse
pmas00002	mas00001_(pTEV5)_forward	mas00002_(pTEV5)_reverse	mas00003_(pWZ21)_forward	mas00004_(pWZ21)_reverse		
pCZ388-dCC-13	CZ-msfGFP-dCC-1-3-F	CZ-msfGFP-dCC-1-3-R				
pCZ382	CZ10944_(pTEV5-bb)_forward	CZ10945_(pTEV5-bb)_reverse	CZ10946_(SNAP)_forward	CZ10947_(SNAP)_(HRSAT)_reverse	CZ10948_(PodJ-1-635)_forward	CZ10949_(PodJ-1-635)_reverse
pCZ479	pWZ24-dCC1-3-F1	pWZ24-dCC1-3-R1				
WZ365	WZH566-pxyfpn-2-Pxyl-sfGFP-PodJ1-635-F	WZH567-pxyfpn-2-Pxyl-sfGFP-PodJ1-635-R				
pSWD259	SWD700_(start)_(sfgfp)_forward	SWD790_(sfgfp)_(HRSAT)_reverse	SWD791_(podJ_251_670)_forward	SWD792_(podJ_251_670)_(stop)_reverse		

Table S2. List of PCR primers

Name	Description
CZ10524_(pSR43_6)_reverse	GCGGCTCTGGATCATTAAAGATTGGCGGATATGTTGGG
CZ10525_(PleC_PAS3)_forward	CCGCCAATCTTTAATGATCCAGAGCCGCAAGGCCG
CZ10583_(CcaS_with_TM)_forward	GACCGCCGCCGACGTCACTGAGCGCCGACGC
CZ10584_(PleC_CD)_reverse	GGCGCTCAGTGACGTCGGCGGCGGTCATGACAAGACC
CZ10596_(pCZ130)_forward	GCAATTTATCTCTTCAAATGTACTAGTGCTTGGATTCTCACCAA
CZ10597_(pCZ130)_reverse	GCTATTTGTTTATTTTTCTTTTTACGGTTCCTGGCCTTTTGC
CZ10598_(CloDF13)_forward	GGCCAGGAACCGTAAAAAGAAAAATAAACAAATAGCTAGCTCACTCGGTCG
CZ10599_(CloDF13)_reverse	GGTGAGAATCCAAGCACTAGTACATTTGAAGAGATAAATTGCACTGAAATC
CZ10622_(Delta_CcaS)_reverse	CCTTGCTCACCATTGCGCCTTCCTCCTATTAAGATTTTAACC
CZ10623_(eyfp)_forward	GGAGGAAGGCGCAATGGTGAGCAAGGGCGAGGAGC
CZ10625_(pCZ10224)_forward	CCGGTCGGCCACCATGATCCAGAGCCGCAAGGC
CZ10626_(eyfp)_reverse	GGCTCTGGATCATGGTGGCCGACCGGTGCTTGTACAGC
CZ10807_(pCDF)_forward	GGTGTGTGGGACGGTGCATCACCGGTCGGCCACCATGG
CZ10808_(pCDF)_reverse	GCACGTTCGTCATCTGGCTGTGGTGATGATGGTGATGGC
CZ10809_(Atu_PleC)_forward	TCACCACAGCCAGATGACGAACGTGCGGCGGG
CZ10810_(Atu_PleC)_reverse	TGGCCGACCGGTGATGCACCGTCCCACACACCCC
CZ10811_(pCDF)_forward	GGCCGCGGTGATGCACCGGTCGGCCACCATGG
CZ10812_(pCDF)_reverse	CGTTGGCGCGTGCCATCTGGCTGTGGTGATGATGGTGATGGC
CZ10813_(Xan_PleC)_forward	TCACCACAGCCAGATGGCACGCGCCAACGCT
CZ10814_(Xan_PleC)_reverse	TGGCCGACCGGTGCATCACCGCGGCCAGCCT
CZ10815_(pCDF)_forward	GCGGGATGTTGCCCACCGGTCGGCCACCATGG
CZ10816_(pCDF)_reverse	CCCCGCGAATCATCTGGCTGTGGTGATGATGGTGATGGC
CZ10817_(Hyp_PleC)_forward	TCACCACAGCCAGATGATTCGCGGGGAAAAGGTAAGGC
CZ10818_(Hyp_PleC)_reverse	TGGCCGACCGGTGGGCAACATCCCGCTGGATGTCG
CZ11031_(pBAD-YFP)_forward	AAGGCGCGCTTGTAAGAAGCTTGGCTGTTTTGGCGG
CZ11032_(pBAD-YFP)_reverse	GACGGGGTGGCCGACCGGTGCTTG
CZ11033_(PSE)_forward	TACAAGCACCGGTCGGCCACCCCGTCGCCGCCGCCCGCG
CZ11034_(PSE)_reverse	AAAACAGCCAAGCTTCTTACAAGCGCGCCTTCGACTTCTTGG
CZ11057_(pWZ21)_forward	GACCGCCGCCGACATCACGGCCATCAAGACCCAGG
CZ11058_(pWZ21)_reverse	GGCTCTGGATCATCAGCAGCAGCGCCAGGGC
CZ11059_(PleC_CD_D_DVT)_forward	GGCGCTGCTGCTGATGATCCAGAGCCGCAAGGCCG
CZ11060_(PleC_CD_D_DVT)_reverse	GGTCTTGATGGCCGTGATGTCGGCGGCGGTCATGACAAGACC
RecUni-1	ATGCCGTTTGTGATGGCTTCCATGTCG
RecXyl-2	TCTTCCGGCAGGAATTCACTCACGCC
WZ0010_(PCDF-YFP)_reverse	GGTGGCCGACCGGTGCTTGTACAGCTCGTCCATGCCG
WZ0011_(HRSAT)_(PODJ)_forward	GACGAGCTGTACAAGCACCGGTCGGCCACCATGACGGCGGCTTCGCCATGG
WZ0024_(mcherry)_reverse	GTCGACCTGCAGGCGCGCCGAGCTCGAATTCTTACTTGTACAGCTCGTCCATGCCGC

WZ0025_(pxyfpc-6)_forward	CTTAGTATATTAGTTAAGTATAAGAAGGAGATATAATGGTGAGCAAGGGCGAGGAGC
WZ0026_(PodJ)_reverse	GTGGCCGGCCGATATCCAATTGAGATCTGCTTAGCGCGCGTAGACCGACAGG
WZ0044_(Plec)_forward	CAGCAGCCATCACCATCATCACCACAGCCAATGGGCAGACACGGGGGGC
WZ0045_(Plec)_reverse	GGTGGCCGACCGGTGGGCCGCCACGAAGTCGCG
WZ0046_(mcherry)_forward	GACTTCGTGGCGGCCCACCGGTCGGCCACCATGG
WZ0050_(PBAD)_forward	GGTCTACGCGCGCTAAGAAGCTTGGCTGTTTTGGCGG
WZ0050_(PBAD)_forward	GGTCTACGCGCGCTAAGAAGCTTGGCTGTTTTGGCGG
WZ0051_(PBAD)_reverse	CGCCCTTGCTCACCATTTAATTCCTCCTGTTAGCCCAAAAA
WZ0052_(YFP-PODJ)_forward	AACAGGAGGAATTAAATGGTGAGCAAGGGCGAGGAGC
WZ0053_(YFP-PODJ)_reverse	AAACAGCCAAGCTTCTTAGCGCGCGTAGACCGACAGG
WZ0173_(pbad)_forward	CGAGCTGTACAAGTAAGAAGCTTGGCTGTTTTGGCGG
WZ0176_(plec-mcherry)_reverse	CCAAAACAGCCAAGCTTCTTACTTGTACAGCTCGTCCATGCCG
WZ0186_(pcdf-mcherry)_reverse	CGAAGCCGCCGTCATGGTGGCCGACCGGTGCTTGTA
WZ0187_(PODJ1-470)_forward	CACCGGTCGGCCACCATGACGGCGGCTTCGCCATGG
WZ0312_(pbad)_reverse	GCGGCTCTGGATCATTTAATTCCTCCTGTTAGCCCAAAAA
WZ0315_(plec-pasc)_forward	CTAACAGGAGGAATTAAATGATCCAGAGCCGCAAGGCCG
WZ0316_(plec-pasc)_reverse	GGTGGCCGACCGGTGGGTGACGTCCAGCGCCACGC
WZ0317_(mcherry)_forward	GCGCTGGACGTCACCCACCGGTCGGCCACCATGGTGAGC
WZ0320_(plec-pasd)_reverse	GGTGGCCGACCGGTGCGTGATGTCGGCGGCGGTCATGACAAGACC
WZ0321_(mcherry)_forward	GCCGCCGACATCACGCACCGGTCGGCCACCATGGTGAGC
WZ0322_(pbad)_reverse	CGATCCGCTCCTCCATTTAATTCCTCCTGTTAGCCCAAAAA
WZ0323_(plec-pasd-atg)_forward	GCTAACAGGAGGAATTAAATGGAGGAGCGGATCGCCCAGG
WZ0324_(pbvmcs-6)_forward	CGAGCTGTACAAGTAAAAAACGGGCCCCCCCTCGAGG
WZ0325_(pbvmcs-6)_reverse	CCCGTGTCTGCCCATCGTTTCCTCGCATCGTGGTTCGG
WZ0326_(plec-mch)_forward	CGATGCGAGGAAACGATGGGCAGACACGGGGGGCC
WZ0327_(plec-mch)_reverse	GGGGGGGCCCGTTTTTTACTTGTACAGCTCGTCCATGCCG
WZ0446_(podjdeltaPSE)_reverse	CAAAACAGCCAAGCTTCTTAGCGCGCGTAGACCGACAGG
CZ-pCZ253-DVT-C-AVA-F	TCGGCGTGGCGCTGGCGGTCGCGGAGGAGCGGATCGCCCAGGCCC
CZ-pCZ253-DVT-C-AVA-R	GACCGCCAGCGCCACGCCGATGATGCGGGCGTGAC
CZ10973_(pTEV5)_forward	GAACTCTACAAATAGTAAGCTAGCCATATGGCCATGGA
CZ10974_(pTEV5)_reverse	CTTCACCTTTAGACATGCCCTGAAAATACAGGTTTTCACTAGTTGGG
CZ10975_(msfGFP)_forward	GTATTTTCAGGGCATGTCTAAAGGTGAAGAACTGTTCACCGGTGTTGT
CZ10976_(msfGFP)_(stop)_reverse	GGCCATATGGCTAGCTTACTATTTGTAGAGTTCATCCATGCCGTGCGTG
mas00001_(pTEV5)_forward	CGAGCTGTACAAGTAATAGGCTAGCCATATGGCCATGGA
mas00002_(pTEV5)_reverse	GCCCTTGCTCACCATGCCCTGAAAATACAGGTTTTCACTAGTTGGG
mas00003_(mCherry)_forward	GTATTTTCAGGGCATGGTGAGCAAGGGCGAGGAGG
mas00004_(mCherry)_reverse	CCATATGGCTAGCCTATTACTTGTACAGCTCGTCCATGCCGC
CZ10944_(pTEV5-bb)_forward	CGAAGGCGCGCTTGTAAGCTAGCCATATGGCCATGGA
CZ10964_(pTEV5-bb)_(S)_reverse	GGTGAACAGTTCTTCACCTTTAGACATGCCCTGAAAATACAGGTTTTCACTAGTTGGG
CZ10965_(msfGFP)_forward	TCAGGGCATGTCTAAAGGTGAAGAACTGTTCACCGGTGTTGT
CZ10966_(msfGFP)_(HRSAT)_reverse	CGAAGCCGCCGTCATGGTGGCCGACCGGTGTTTGTAGAGTTCATCCATGCCGTGCGT

CZ10948_(PodJ-1-635)_forward	CCGGTCGGCCACCATGACGGCGGCTTCGCCA
CZ10949_(PodJ-1-635)_reverse	TATGGCTAGCTTACAAGCGCGCCTTCGACTTCTTGGT
mas00001_(pTEV5)_forward	GGACGAGCTGTACAAGTAGGCTAGCCATATGGCCATGGA
mas00002_(pTEV5)_reverse	CGACCTCGGCCTTGCCCTGAAAATACAGGTTTTCACTAGTTGGG
mas00003_(pWZ21)_forward	CCTGTATTTTCAGGGCAAGGCCGAGGTCGCCCATCG
mas00004_(pWZ21)_reverse	GCCATATGGCTAGCCTACTTGTACAGCTCGTCCATGCCGCC
CZ-msfGFP-dCC-1-3-F	ACAAACACCGGTCGGCCACCTTGGGCGCCGTCGAGACTGCCAATC
CZ-msfGFP-dCC-1-3-R	GGTGGCCGACCGGTGTTTGTAGAGTTCATCCATGCCGTGCGT
CZ10944_(pTEV5-bb)_forward	CGAAGGCGCGCTTGTAAGCTAGCCATATGGCCATGGA
CZ10945_(pTEV5-bb)_reverse	CGCAATCTTTGTCCATGCCCTGAAAATACAGGTTTTCACTAGTTGGG
CZ10946_(SNAP)_forward	TTTTCAGGGCATGGACAAAGATTGCGAAATGAAACGTACCACCC
CZ10947_(SNAP)_(HRSAT)_reverse	CGAAGCCGCCGTCATGGTGGCCGACCGGTGGGATCCTGGCGCGCCTATACC
CZ10948_(PodJ-1-635)_forward	CCGGTCGGCCACCATGACGGCGGCTTCGCCA
CZ10949_(PodJ-1-635)_reverse	TATGGCTAGCTTACAAGCGCGCCTTCGACTTCTTGGT
pWZ24-dCC1-3-F1	TTGGGCGCCGTCGAGACTGCCAATCCCGCCACGGG
pWZ24-dCC1-3-R1	GGCAGTCTCGACGGCGCCCAAGGTGGCCGACCGGTGCTTGTACAG
WZH566-pxyfpn-2-Pxyl-sfGFP-PodJ1-635-F	GGCGCGCTTGTAAGCCTTAATTAATATGCA
WZH567-pxyfpn-2-Pxyl-sfGFP-PodJ1-635-R	TTAAGGCTTACAAGCGCGCCTTCGACTTCT
SWD700_(start)_(sfgfp)_forward	ATATGCATGGTACCTTAAGATCTCGAATGAGCAAAGGAGAAGAACTTTTCACTGG
SWD790_(sfgfp)_(HRSAT)_reverse	GCCCAAGGTGGCCGACCGGTGTTTGTAGAGCTCATCCATGCC
SWD791_(podJ_251_670)_forward	TACAAACACCGGTCGGCCACCTTGGGCGCCGTCGAGACTG
SWD792_(podJ_251_670)_(stop)_reverse	ACTAGTGGATCCCCCGGGCTGCAGCTTCAGCCCAAGCGCGCCTTCGAC

Table S3. List of plasmids constructed

Plasmid	Description	Reference
pTEV5	bacterial expression vector	Rocco 2008
pTEV6	bacterial expression vector with MBP solubilization tag	Rocco 2008
pXCHYC-6	*C. crescentus* integrating C-terminal mCherry fusion vector	Thanbickler paper
pBXMCS-2	*C. crescentus* high-copy replicating C-terminal M2 fusion vector	Thanbickler paper
pWZ21	pACYC-PleC-mCherry	this study
pWZ24	pBAD-YFP-PodJ	this study
pWZ12	pCDF-YFP-PodJ	this study
podj02	pCDF-YFP-PodJ(1-702)	this study
podj17	pCDF--YFP-PodJ(1-470, 643-702)	this study
podj23	DH5α pCDF-YFP-PodJ(1-635)	this study
podj31-7	pCDF-YFP-PodJ(1-588, 643-702)	this study
podj33	pCDF-YFP-PodJ(1-470, 589-702)	this study
podj32	pCDF-YFP-PodJ(1-470, PopZ 24-102, 643-702)	this study
pWZ138-kan	pBVMCS-6-Pvan-Plec-mCherry	this study
pWZ138-chlor	pBVMCS-2-Pvan-Plec-mCherry	this study
pCZ340	pCDF-PleC(Atu)-CFP	this study
pCZ341	pCDF-PleC(Xan)-CFP	this study
pCZ342	pCDF-PleC(Hyp)-CFP	this study
plec2	pBAD-PleC(1-53, 302-842)-mcherry	this study
plec3	pBAD-PleC(1-301, 551-842)-mcherry	this study
plec4	pBAD-PleC(1-550)-mcherry	this study
pWZ127	pBAD-PleC-PAS C-mcherry	this study
pWZ135	pBAD-PleC-PAS D-mcherry	this study
pWZ136	pBAD-PleC-PAS CD-mcherry	this study
pBAD-PleC-delta PAS C	pBAD-PleC-delta PAS C-mcherry	this study
pBAD-PleC-delta PAS D	pBAD-PleC-delta PAS D-mcherry	this study
YFP-J23102-pCZ267	pACYCDuet-J23102-YFP-PleC(302-548)-CcaS(502-753)	this study
pCZ259	pProTet.E333-CcaR	this study
pCZ253	pACYCDuet-CcaS(1-57)-PleC(302-548)-CcaS(502-753)	this study
pW173	pBAD-YFP-PodJ(1-470, 636-974)	this study
pCZ412	pBAD-YFP-PodJ(470-635)	this study
pWZ24-dPeri	pBAD-YFP-PodJ(1-702)	this study
pBAD vector	pBAD empty vector	this study
pCZ253 (D433A, T435A)	pACYCDuet-CcaS(1-57)-PleC(302-548, D433A, T435A)-CcaS(502-753)	this study
pCZ253 (D548A, I549V, T550A)	pACYCDuet-CcaS(1-57)-PleC(302-548, D548A, I549V, T550A)-CcaS(502-753)	this study

pCZ253 (D433A, T435A, D548A, I549V, T550A)	pACYCDuet-CcaS(1-57)-PleC(302-548, D433A, T435A, D548A, I549V, T550A)-CcaS(502-753)	this study
pCZ391	pTEV5-sfGFP	this study
pmas00001	pTEV5-mCherry	this study
pCZ388	pTEV5-sfGFP-PodJ 1-635	this study
pmas00002	pTEV5-PleC-AB-HK-mCherry	this study
pCZ388-dCC-13	pTEV5-sfGFP-PodJ 250-635	this study
pCZ382	pTEV5-SNAP-PodJ 1--635	this study
pCZ479	pBAD-YFP-PodJ 250-635	this study
WZ365	pxyfpn-2-Pxyl-sfGFP-PodJ1-635	this study
pSWD259	pXyl-2-sfgfp-hrsat-podJ(251-635)	this study

Table S4. List of all strains used in this study

Strain	Description	Plasmid Number	Reference
E. coli DH5α	bacterial cloning strain		Invitrogen
E. coli BL21	bacterial expression strain		Novagen
BW29655	bacterial strain for reporter gene assay		
C. crescentus NA1000	laboratory *Caulobacter crescentus* strain		
WSC1292	DH5α pACYC-PleC-mCherry	pWZ21	this study
WSC1347	BL21(DE3) pACYC-PleC-mCherry	pWZ21	this study
WSC1234	DH5α pBAD-YFP-PodJ	pWZ24	this study
CZ668	BL21(DE3) pBAD-YFP-PodJ	pWZ24	this study
WSC1232	DH5α pCDF-YFP-PodJ	pWZ12	this study
WSC1343	BL21(DE3) pCDF-YFP-PodJ	pWZ12	this study
WSC1354	BL21(DE3) pCDF-YFP-PodJ, pACYC-PleC-mCherry	pWZ12, pWZ21	this study

WSC1237	DH5α pCDF-YFP-PodJ(1-702)	pod02	this study
WSC1367	BL21(DE3) pCDF-YFP-PodJ(1-702)	pod02	this study
WSC1258	DH5α pCDF-YFP-PodJ(1-635)	podj23	this study
WSC1252	DH5α pCDF--YFP-PodJ(1-470, 643-702)	podj17	this study
WSC1344	BL21(DE3) pCDF--YFP-PodJ(1-470, 643-702)	podj17	this study
WSC1266	DH5α pCDF-YFP-PodJ(1-588, 643-702)	podj31-7	this study
WSC1341	BL21(DE3) pCDF-YFP-PodJ(1-588, 643-702)	podj31-7	this study
WSC1348	BL21(DE3) pCDF-YFP-PodJ(1-588, 643-702), pACYC-PleC-mCherry	pod31-7, pWZ21	this study
WSC1268	DH5α pCDF-YFP-PodJ(1-470, 589-702)	podj33	this study
WSC1342	BL21(DE3) pCDF-YFP-PodJ(1-470, 589-702)	podj33	this study
WSC1351	BL21(DE3) pCDF-YFP-PodJ(1-470, 589-702), pACYC-Plec-mcherry	pod33, pWZ21	this study
WSC1267	DH5α pCDF-YFP-PodJ(1-470, PopZ 24-102, 643-702)	podj32	this study

WSC1208	NA1000 pBVMCS-6-Pvan-Plec-mCherry	pWZ138	this study
WSC1209	DpodJ, pBVMCS-6-Pvan-Plec-mCherry	pWZ138	this study
WSC1210	Delta podJ, pBVMCS-6-Pvan-Plec-mCherry, pXYFPN-2-Pxyl-sfGFP-PodJ	pWZ138	this study
WSC1221	Delta podJ, pXYFPN-2-Pxyl-sfGFP-PodJΔ471-635, pBVMCS-6-Pvan-Plec-mCherry	pWZ138	this study
CZ588	BL21(DE3) pCDF-PleC(Atu)-CFP	pCZ340	this study
CZ565	BL21(DE3) pBAD-YFP-PodJ, pCDF-PleC(Atu)-CFP	pWZ24, pC340	this study
CZ589	BL21(DE3) pCDF-PleC(Xan)-CFP	pCZ341	this study
CZ564	BL21(DE3) pBAD-YFP-PodJ, pCDF-PleC(Xan)-CFP	pWZ24, pC341	this study
CZ590	BL21(DE3) pCDF-PleC(Hyp)-CFP	pCZ342	this study
CZ563	BL21(DE3) pBAD-YFP-PodJ, pCDF-PleC(Hyp)-CFP	pWZ24, pC342	this study
WSC1297	DH5α pBAD-PleC(1-53, 302-842)-mcherry	plec2	this study
WSC1298	DH5α pBAD-PleC(1-301, 551-842)-mcherry	plec3	this study

WSC1299	DH5α pBAD-PleC(1-550)-mcherry	plec4	this study
WSC1300	DH5α pBAD-PleC-PAS A-mcherry	pWZ127	this study
WSC1301	DH5α pBAD-PleC-PAS B-mcherry	pWZ135	this study
WSC1302	DH5α pBAD-PleC-PAS AB-mcherry	pWZ136	this study
WSC1303	DH5α pBAD-PleC-delta PAS A-mcherry	pBAD-PleC-delta PAS C	this study
WSC1304	BL21(DE3) pBAD-PleC-delta PAS B-mcherry	pBAD-PleC-delta PAS D	this study
WSC1210	Delta PodJ, pBVMCS-6-Pvan-PleC-mCh, pXYFPN-2-Pxyl-sfGFP-PodJ	pWZ138	this study
CZ446	BW29655, pACYCDuet-J23102-YFP-PleC(302-548)-CcaS(502-753)	YFP-J23102-pCZ267	this study
CZ442	BW29655, pACYCDuet-J23102-YFP-PleC(302-548)-CcaS(502-753), pBAD-CFP-PodJ,pProTet.E333-CcaR	YFP-J23102-pCZ267, pWZ74, pCZ259	this study
CZ388	BW29655, pACYCDuet-CcaS(1-57)-PleC(302-548)-CcaS(502-	pCZ253, pWZ24,	this study

	753), pBAD-YFP-PodJ,pProTet.E333-CcaR	pCZ259	
CZ145	BW29655, pACYCDuet-CcaS(1-57)-PleC(302-548)-CcaS(502-753), pBAD-YFP-PodJ(1-470, 636-974),pProTet.E333-CcaR	pCZ253, pW173, pCZ259	this study
CZ84	BW29655, pACYCDuet-CcaS(1-57)-PleC(302-548)-CcaS(502-753), pBAD-YFP-PodJ(470-635),pProTet.E333-CcaR	pCZ253, pCZ412, pCZ259	this study
CZ639	BW29655, pACYCDuet-CcaS(1-57)-PleC(302-548)-CcaS(502-753), pBAD-YFP-PodJ(1-702),pProTet.E333-CcaR	pCZ253, pWZ24-dPeri, pCZ259	this study
CZ612	BW29655, pACYCDuet-CcaS(1-57)-PleC(302-548)-CcaS(502-753), pBAD vector,pProTet.E333-CcaR	pCZ253, pBAD vector, pCZ259	this study
CZ619	BW29655, pACYCDuet-CcaS(1-57)-PleC(302-548, D433A, T435A)-CcaS(502-753), pBAD-YFP-PodJ,pProTet.E333-CcaR	pCZ253(D433A, T435A), pWZ24, pCZ259	this study
CZ704	BW29655, pACYCDuet-CcaS(1-57)-PleC(302-548, D548A, I549V, T550A)-CcaS(502-753), pBAD-YFP-PodJ,pProTet.E333-CcaR	pCZ253(D548A, I549V, T550A), pWZ24, pCZ259	this study
CZ706	BW29655, pACYCDuet-CcaS(1-57)-PleC(302-548, D433A, T435A, D548A, I549V, T550A)-CcaS(502-753), pBAD-YFP-	pCZ253(D346A, D348A, D433A,	this study

	PodJ,pProTet.E333-CcaR	D435A), pWZ24,pCZ259	
CZ633	BW29655, pACYCDuet-CcaS(1-57)-PleC(302-548, D433A, T435A)-CcaS(502-753), pBAD-YFP-PodJ,pProTet.E333-CcaR	pCZ253(D433A, T435A), pBAD vector, pCZ259	this study
CZ634	BW29655, pACYCDuet-CcaS(1-57)-PleC(302-548, D433A, T435A, D548A, I549V, T550A)-CcaS(502-753), pBAD-YFP-PodJ,pProTet.E333-CcaR	pCZ253(D346A, D348A, D433A, D435A), pBAD vector, pCZ259	this study
KAK465	NA1000, pBVMCS-2	pBVMCS-2	this study
KAK466	Delta PleC, pBVMCS-2	pBVMCS-2	this study
KAK343	Delta PleC, pBVMCS-6-PleC-mCherry	pBVMCS-6-PleC-mCherry	this study
KAK344	Delta PleC, pBVMCS-6-PleC delta AB-mCherry	pBVMCS-6-PleC delta CD-mCherry	this study
KAK345	Delta PleC, pBVMCS-6-PleC delta A-mCherry	pBVMCS-6-PleC	this study

		delta C-mCherry	
KAK346	Delta PleC, pBVMCS-6-PleC delta B-mCherry	pBVMCS-6-PleC delta D-mCherry	this study
MJC120	Delta PleC pBVMCS-6	pBVMCS-6	this study
MJC122	NA1000 PleC pBVMCS-6	pBVMCS-6	this study
CZ683	Delta PleC, pBVMCS-6-PleC -mCherry	pWZ138-kan	this study
CZ349	DH5α pTEV5-sfGFP	pCZ391	this study
CZ149	Rosetta pTEV5-mCherry	pmas00001	this study
CZ604	BL21, pTEV5-sfGFP-PodJ 1-635	pCZ388	this study
CZ147	Rosetta pTEV5-PleC-AB-HK-mCherry	pmas00001	this study
CZ703	Rosetta, pTEV5-sfGFP-PodJ 250-635	pCZ388-dCC-13	this study
CZ602	BL21, pTEV5-SNAP-PodJ 1--635	pCZ382	this study
WSC0398	Rosetta, pET28-His-PopZ		this study

WSC1325	BL21, PTEV5-CCKA-cc		this study
WSC1326	BL21, PTEV5-DIVL-cc		this study
CZ718	BW29655, pACYCDuet-CcaS(1-57)-PleC(302-548)-CcaS(502-753), pBAD-YFP-PodJ 250-635,pProTet.E333-CcaR	pCZ253, pCZ479, pCZ259	this study
SWD655	NA1000, podJ::chlor PpopZ::mCherry-PopZ	WZ365	this study
SWD649	NA1000, podJ::chlor	pSWD259	this study

Text S1

Construction of plasmids

The plasmids used in this study can be found in the Plasmids section Table S3. Below is an example of the generation of plasmid using the Gibson assembly approach based on Table S3 information. This can also be similarly applied to construct other plasmids used in this study. Oligonucleotide primers applied for amplification of the gene insert are designed using the j5 online program, and they featured overlaps of 26 bases to the insertion site in the plasmid.[10]. Oligonucleotides were synthesized by IDT (Coralville, IA), and all DNA sequencing reactions were performed by Genewiz (South Plainfield, NJ). A Gibson reaction master mix was prepared from 5x reaction buffer, T5 exonuclease (NEB), Phusion polymerase (NEB), Taq ligase (NEB), and stored as aliquots of 15 μl at -20°C[11]. The plasmid pCZ253 is designed for the expression of chimera sensor PleC-CcaS based on the backbone of pACYCDuet with the fusion of CcaS(1-57)-PleC(302-548)-CcaS(502-753) driven by a constitutive promoter. Amino acids 1-57 of CcaS are fused to 302-548 of PleC, followed by residues 502-753 from CcaS. The vector and insert were linearized through PCR using standard protocols for Phusion polymerase described in the plasmid cloning strategies section with primer pairs of CZ583/CZ524 and CZ525/CZ584, respectively. PCR products with 26 base pair overhangs for Gibson assembly were constructed following a molar ratio of linearized vector: insert = 1:10 (at least 100ng for the vector). An annealing temperature of 55°C for 1h was used, followed by 10 min at 4°C and 10 μl were then transformed into chemically competent *E. coli* DH5a cells using the KCM transformation method and plated on LB/agar supplemented with the appropriate antibiotic. Colonies were

screened using primers CZ525/CZ584sing DreamTaq PCR with a 1-minute extension time.

Reference:

1. Curtis, P.D. et al. The scaffolding and signaling functions of a localization factor impact polar development. *Mol Microbiol* **84**, 712-35 (2012).
2. Lawler, M.L., Larson, D.E., Hinz, A.J., Klein, D. & Brun, Y.V. Dissection of functional domains of the polar localization factor PodJ in Caulobacter crescentus. *Mol Microbiol* **59**, 301-16 (2006).
3. Jones, D.T. Protein secondary structure prediction based on position-specific scoring matrices. *J Mol Biol* **292**, 195-202 (1999).
4. Fiser, A. & Sali, A. Modeller: generation and refinement of homology-based protein structure models. *Methods Enzymol* **374**, 461-91 (2003).
5. Emenecker, R.J., Griffith, D. & Holehouse, A.S. metapredict: a fast, accurate, and easy-to-use cross-platform predictor of consensus disorder. *bioRxiv*, 2021.05.30.446349 (2021).
6. UniProt, C. UniProt: the universal protein knowledgebase in 2021. *Nucleic Acids Res* **49**, D480-D489 (2021).
7. Hofmann K. and Stoffel, W. TMbase-a database of membrane spanning proteins segments. *Biol. Chem. Hoppe Seyler* **347**, 166 (1993).
8. Rice, P., Longden, I. & Bleasby, A. EMBOSS: the European Molecular Biology Open Software Suite. *Trends Genet* **16**, 276-7 (2000).
9. Crooks, G.E., Hon, G., Chandonia, J.M. & Brenner, S.E. WebLogo: a sequence logo generator. *Genome Res* **14**, 1188-90 (2004).
10. Hillson, N.J., Rosengarten, R.D. & Keasling, J.D. j5 DNA assembly design automation software. *ACS Synth Biol* **1**, 14-21 (2012).
11. Gibson, D.G. et al. Enzymatic assembly of DNA molecules up to several hundred kilobases. *Nat Methods* **6**, 343-5 (2009).
